# Supplementary material for: Relationship between saturation and contrast during internal limiting membrane peeling in 3D digital vitreoretinal surgery: A retrospective study
Source: PLoS One. 2025 Dec 3;20(12):e0337416. doi: 10.1371/journal.pone.0337416 (PMC12674541; doi:10.1371/journal.pone.0337416)
Supplement: Attachment — Submitted filename: Response to Reviewers.docx [file pone.0337416.s003.docx]

**Date:** October 21, 2025
**Manuscript ID:** PONE-D-25-33091
**Corresponding Author:** Yoshihito Sakanishi
**Email:** ysakani@juntendo.ac.jp

**Re: Resubmission of revised manuscript—"Relationship between saturation and contrast during internal limiting membrane peeling in 3D digital vitreoretinal surgery: A retrospective study"**

**Dr. Koichi Nishitsuka**
Academic Editor
PLOS ONE
San Francisco, CA, USA

**RESPONSE TO REVIEWERS**

Dear Dr. Nishitsuka,

Thank you for your constructive feedback and the opportunity to revise and resubmit our manuscript. We have carefully addressed all the points raised by the editor and reviewer, and we believe these revisions have significantly strengthened our work. Below, we provide detailed point-by-point responses to each comment, along with specific information about the changes made in the revised manuscript.

**JOURNAL REQUIREMENTS**

**Requirement 1: PLOS ONE Style Requirements**

**Response:** We have reviewed and ensured that our manuscript meets all PLOS ONE style requirements, including formatting guidelines, file naming conventions, and structural　requirements as specified in the PLOS ONE style templates.

**Changes made:** We have verified manuscript formatting compliance with PLOS ONE guidelines, confirmed proper file naming conventions, and ensured adherence to the required document structure and citation style.

**Requirement 2: Participant Consent Details**

**Response:** We have provided comprehensive details regarding participant consent procedures, including the specific type of consent obtained and the ethical approval process. The retrospective nature of the study warranted an opt-out consent process, which was approved by our institutional ethics committee.

**Changes made:** Methods section - Added complete ethical statement and consent procedure details:

"This retrospective study was conducted in accordance with the Declaration of Helsinki. In addition, the ethics committee of the Juntendo Clinical Research and Trials Center approved the study (approval number, E21-0180). We accessed the medical records on November 2, 2023, for research purposes, and all extracted patient data were anonymized for analysis. Patient consent was obtained through an opt-out process approved by the ethics committee. Patients were informed about the research through the hospital homepage and could withdraw consent any time. Written informed consent was waived due to the retrospective observational nature of the study, as approved by the ethics committee."

**Requirement 3: Data Availability Statement (Critical Requirement)**

**Response:** We confirm that our submission contains data required to replicate the results of our study. The minimal dataset, includes contrast ratio measurements　and surgical parameters necessary to reproduce our findings.

**Requirement 5: Reference List Review**

**Response:** We have thoroughly reviewed our reference list to ensure completeness and accuracy. All citations have been verified for proper formatting and relevance to the study content.

**EDITOR COMMENTS**

**Editor Comment 1:**

"The analysis of hemorrhage detection is based on only a few cases and should be considered preliminary. Please acknowledge this explicitly in the Discussion as a limitation."

**Response:** We agree with this important concern regarding the limited sample size for hemorrhage detection analysis. We have explicitly acknowledged this limitation and emphasized the preliminary nature of these findings in our Discussion section.

**Changes made:** Discussion section, Limitations paragraph - Added text:

"First, the analysis of hemorrhage detection is based on the small number of cases and should be considered preliminary."

**Editor Comment 2:**

"Clinical outcomes such as surgical performance or postoperative vision were not assessed, which limits the study to proof-of-concept. Please add a statement to this effect in the Discussion."

**Response:** We acknowledge that our study did not assess clinical outcomes and have explicitly stated that this work should be considered a proof-of-concept study demonstrating technical feasibility rather than clinical superiority.

**Changes made:** Discussion section, Limitations paragraph - Added text:

"Second, clinical outcomes such as actual surgical performance metrics (e.g., ILM peeling time, ease of procedure, complication rates) or postoperative visual outcomes were not assessed in this study. Therefore, this work should be considered a proof-of-concept study demonstrating the technical feasibility of contrast enhancement rather than evidence of clinical superiority."

**Editor Comment 3:**

"Contrast measurements were performed by two evaluators, but inter-observer variability was not statistically assessed. Please note this in the limitations section."

**Response:** We have acknowledged this limitation and noted that the lack of formal inter-observer variability assessment may affect the reproducibility of our contrast measurements.

**Changes made:** Discussion section, Limitations paragraph - Added text:

"Third, contrast measurements were performed by only two evaluators, and interobserver variability was not statistically assessed through formal reliability testing. This may affect the reproducibility of our contrast measurements."

**REVIEWER #1 COMMENTS**

**Reviewer Comment 1:**

"Although not the primary aim of this study, the authors could suggest that future research evaluate actual surgical performance or clinical outcomes (e.g., ILM peeling time, ease of procedure, complication rates, postoperative vision)."

**Response:** We appreciate this excellent suggestion and have incorporated specific directions for future research that include clinical outcome measures to validate our technical findings.

**Changes made:** Conclusion section - Added text:

"Further large multicenter cohort studies with clinical outcome measures are required to validate the present findings and establish the clinical benefits of these image enhancement techniques."

**Reviewer Comment 2:**

"The analysis of hemorrhage detection appears to rely on a limited number of cases; statistical robustness is therefore limited. Moreover, no quantitative data on the frequency or severity of hemorrhage across groups are provided."

**Response:** We acknowledge this limitation regarding the statistical robustness of our hemorrhage detection analysis. This concern has been addressed through the addition of explicit acknowledgment of the preliminary nature of these findings.

**Changes made:** Discussion section, Limitations paragraph - (Addressed through the same addition as Editor Comment 1 response, acknowledging the preliminary nature of hemorrhage detection analysis based on small sample size.)

**Reviewer Comment 3:**

"The study is restricted to NGENUITY version 1.4, and the findings may not necessarily be generalizable to newer versions or other visualization platforms."

**Response:** We fully agree with this important limitation regarding generalizability. We have acknowledged that our findings are specific to NGENUITY version 1.4 and have noted the need for validation across different systems and software versions.

**Changes made:** Discussion section, Limitations paragraph - Added text:

"Fifth, this study is limited to NGENUITY version 1.4, and the findings may not be generalizable to newer versions. Future studies should validate these findings across different systems and software versions. The NGENUITY ver1.5 is equipped with a mode that emphasizes blue even in the non-monochromatic mode, which will need to be considered in future studies."

**Reviewer Comment 4:**

"Contrast measurements were performed by only two evaluators, and potential inter-observer variability was not fully assessed."

**Response:** This important methodological limitation has been addressed in our revised manuscript through explicit acknowledgment in the limitations section.

**Changes made:** Discussion section, Limitations paragraph - (Addressed through the same addition as Editor Comment 3 response, noting the lack of statistical assessment of inter-observer variability.)

**SUMMARY OF REVISIONS**

We have made comprehensive revisions to address all concerns raised during the review process. The key changes include:

- Enhanced ethical statement with detailed consent procedures in the Methods section
- Updated Data Availability Statement clarifying minimal dataset accessibility and content
- Expanded limitations section addressing hemorrhage detection sample size, clinical outcome assessment, inter-observer variability, and NGENUITY version generalizability
- Added future research directions in the Conclusion section
- Verified compliance with all PLOS ONE formatting and style requirements

**Files Submitted:**

- Response to Reviewers (this document)
- Revised Manuscript with Track Changes
- Clean Revised Manuscript

We believe these revisions have significantly strengthened the manuscript and address all concerns raised during the review process. We are grateful for the thorough and constructive feedback that has helped improve our work.

Sincerely,

**Yoshihito Sakanishi, MD, PhD**
Corresponding Author
Department of Ophthalmology
Juntendo University Urayasu Hospital
2-1-1 Tomioka, Urayasu, Chiba 279-0021, Japan
Email: ysakani@juntendo.ac.jp

On behalf of all co-authors:
Saaya Fukase, Keiji Matsumoto, Ayumi Usui-Ouchi, Shintaro Nakao, Nobuyuki Ebihara
